# “Getting Autism”: Educators, Parents, and Autistic Adults and Teens Reflect on the Importance of Teachers Who Understand

**DOI:** 10.1007/s10803-024-06353-y

**Published:** 2024-05-14

**Authors:** Connie Anderson, Caroline I. Wood, Leah Franklin, Alan Iampieri, Clare Sarsony

**Affiliations:** 1 Department of Health Sciences, Towson University, 8000 York Road–CHP Dean’s Office, Towson, MD 21252 USA; 2 Department of English (former Research Assistant on the project), Towson University, 8000 York Road, Towson, MD 21252 USA; 3 Department of Psychology (former Research Assistant on the project), Towson University, 8000 York Road, Towson, MD 21252 USA; 4 Former Research Assistant, Currently Unaffiliated, Silver Spring, MD USA

**Keywords:** Autism, Young adults, Adolescents, School experiences, Special education, Qualitative methods

## Abstract

**Purpose:**

To explore the perspectives of educators, parents, and individuals on the autism spectrum regarding the qualities of teachers best equipped to support autistic students.

**Methods:**

In qualitative interviews parents of autistic adults (*n* = 35) discussed experiences they and their child faced during the school years, as did young autistic adults (*n* = 12) and teens (*n* = 11). Nineteen educators were also interviewed regarding how autistic students and their teachers were faring in public schools as well as what qualities and skills teachers needed to best support these students.

**Results:**

A critical emergent theme was the importance of educators who possess a deep, intuitive understanding of autistic individuals. “Getting autism” involved autism knowledge, obtained through training or experience; an intuitive ability to read, respond to, and appreciate autistic students; and flexibility adapting to these students’ needs. Autistic students, parents, and educators alike experienced a stigmatizing judgement and lack of support from unenlightened individuals in the schools. In contrast, there was great appreciation for those who “got autism” and were able to ease the way of autistic students, or support autism-friendly teachers, in whatever grade or situation.

**Conclusion:**

Research investigating how to cultivate, support, and reward autism-affirming teachers is needed. This will likely involve both didactic and experiential autism-focused training as well as recognition of the importance of high emotional intelligence and other qualities of teachers who “get autism.” Future research should also explore the effects on these teachers and their students of larger systems, policies, and practices.

## Introduction

Most autistic children and teens, like other children and teens, spend a great deal of their time in school: at least 180 days per year in most U.S. states (Education Commission of the States, 2023). It is where they receive not only academic instruction, but interventions, socialization, and hopefully a sense of community. How school personnel, especially teachers, contribute to positive experiences that build towards future well-being and success or negative ones that undermine them is a question of critical interest.

Autistic students journeying through their school years, together with their parents, encounter general education teachers, special education teachers, guidance counselors, school psychologists, transition specialists, and teaching assistants, to name a few. These professionals’ understanding of autism and their ability to support students on the autism spectrum vary greatly. At the same time, these students are themselves anything but heterogeneous despite two core commonalities: (1) an inability to read and respond to the social world in an intuitive way; and (2) the presence of restricted, repetitive behaviors activities, and interests (American Psychiatric Association, 2022).

Each autistic student brings a unique combination of challenges and strengths to school. First, cognitive ability varies widely (American Psychiatric Association, 2022). According to Maenner et al. (2023), roughly 38% of 8-year-old autistic children in the U.S. are intellectually disabled (IQ ≤ 70), with about 39% in the average or high range (IQ > 85) and another 23% in between. Social motivation likewise varies, with a number of individuals eager to socially connect while others are socially disinterested (Scheeren et al., 2012). Autistic students may communicate verbally or employ assistive technology, signing, or other alternatives (van der Meer et al., 2011). Many have executive functioning issues and the disorganization and frustration that come with them (Monteiro, 2021). Co-occurring conditions are common, occur in endless combinations, and include (but are not limited to) seizures, attention deficit hyperactivity disorder (ADHD), anxiety (Shoaib et al., 2022), gender dysphoria (Kallitsounaki & Williams, 2023) and eating disorders (Brede et al., 2020). In sum, autistic students’ needs are varied and multifaceted.

The educators who currently assist these students play a role with roots going back at least to the ancient Greeks, one that has morphed over time and across societies, including beliefs regarding what should be taught, to what purpose (indoctrination vs. critical thinking), whether punishment or play best motivates students, and who should receive instruction (Thomas, 2021). Regarding this last, it should be noted that people with disabilities were denied access until very recently. Children with disabilities in the United States were not guaranteed a public education until the 1975 passage of the Education for All Handicapped Children Act, later renamed the Individuals with Disabilities Education Act (IDEA, 2012). Before that, they had no right to attend public school at all, with some states outlawing their participation while others left it up to individual schools to decide. (Individuals with Disabilities Education Act, 2023).

Special education now takes place within public schools that have a daunting responsibility: To educate and socialize a society’s children from pre-school to high school. They not only provide access to instruction, but to athletics and the arts while managing programs for those who are English language learners, are gifted, or have disabilities (National Center for Education Statistics, 2022; National Center for Education Statistics, 2023a). There may be a nurse providing health care, a social worker dealing with mental health and family issues, and food services providing breakfast or lunch for students from low-income households (Keppler, 2020). Schools are generally required to fulfill these missions without adequate resources (Allegretto et al., 2022) due in part to a nearly $150 billion annual shortfall in state- and district-provided funds (The Century Foundation, 2020). Neither can the schools depend on the federal government to make up this deficit. Although special education is one of the few federally funded priorities in local schools, the federal government has never provided “full funding” (Congressional Research Service, 2019, p. 21) –an amount for each special needs student equivalent to “40% of the average per-pupil expenditure (APPE) in public elementary schools and secondary schools in the United States” (IDEA, 2012 – Sect. 1411, p. 861). For example, in FY2019 federal funds appropriated for special needs students were equivalent to only 14.3% of the APPE (Congressional Research Service, 2019). Whether this is due to political conflict over priorities at the national level, steeped in clashing attitudes about the worth of public education and the value of people with disabilities (Blad, 2020), the result is that local school districts, especially those in low-income areas with a weak tax base, are hard pressed to provide adequate special education services (Kolbe et al., 2023). This leaves schools struggling to meet their legal obligations to special education students as evidenced by the fact that 37 of 60 (62%) U. S. states and territories recently failed to meet legal requirements for serving students with disabilities ages 3 through 21 (Individuals with Disabilities Education Act, 2021).

According to the National Center for Education Statistics (2023b), 7.3 million students or about 15% of all those attending public school were served under IDEA in school year 2021-22. Of these, 12% or approximately 880,000 had autism. As of Fall 2021, most autistic students were in regular public schools, with 40.8% included inside a general classroom 80% or more of the time, 17.2% included 40–79% of the time, and 34.1% included less than 40% of the time. The remainder were in a separate day school for children with disabilities (6%), private school (1.1%), a separate residential facility (0.3%), or on Home Hospital (0.3%) (National Center for Education Statistics, 2023c). Clearly, practices and relationships within public schools will have a major impact on the lives of most autistic students.

The current study focuses on two key questions: Against this complex legal, social, and organizational background, what is it that makes an individual educator encountering autistic students and their families able to connect with and support them? What is lacking when they cannot?

## Methods

As part of a project focused on the transition to adulthood, qualitative interviews were conducted in 2015–2017 with 35 parents of 36 young autistic adults (YAAs). (One family had two sons on the autism spectrum.) These featured open-ended questions about the family’s life with an intentional emphasis on transition issues: postsecondary education, employment, and adult services. Unexpectedly, many early interviewed parents offered recollections of their adult child’s school-based experiences – so much so that we began to ask questions about this topic explicitly. Such generation of new questions mid-analysis is part of a grounded theory approach, specifically “theoretical sampling,” wherein researchers may “collect data from places, people, and events that will maximize opportunities to develop concepts in terms of their properties and dimensions, uncover variations, and identify relationships between concepts” (Corbin & Strauss, 2015, p. 134).

Next, we interviewed 19 educators of autistic students (2019). Our intent was to employ “data triangulation” by interviewing people occupying different roles within the same institution (Denzin, 1970, p. 301). As Stake (2005) put it: “The qualitative researcher is interested in diversity of perception, even the multiple realities within which people live. Triangulation helps to identify different realities” (p. 454).

Like their parents, some of the 12 YAAs interviewed shared comments about their past school-based experiences, including three who mentioned past teachers. To enhance autistic voices, we included in our analysis school-focused material from interviews we conducted in 2020–2021 with 11 teens on the autism spectrum recruited via the Simons Foundation Powering Autism Research for Knowledge (SPARK) Research Match program (The SPARK Consortium, 2018). (There had been 13 teens, but two who had attended private school exclusively were excluded.) Those interviews, with families from across the United States, focused on motivations for research participation, not school, but like the YAAs the teens broached school-based experiences and all but one mentioned relationships with teachers. (For a detailed description of this study, see Anderson et al., 2022). Together, YAA and teen recollections hinted at what went right or wrong in the schools for these students.

The Towson University Institutional Review Board (IRB) approved parent (#15-X015), YAA (#1611009343), educator (#1801028959), and teen (#1141) protocols. Informed consent was received from YAAs and their parents, educators, and the parents of teens, while teens provided assent.

Material gathered was voluminous, meriting investigation at multiple levels. A useful theoretical framework for conceptualizing this is Bronfenbrenner’s pioneering Socioecological Model (1977) as modified by McLeroy et al. (1988). At the heart of this model is a nested or telescoping perspective that permits levels of analysis from the individual through society-as-a-whole as well as an acknowledgement that these interact and influence one another. As envisioned by McLeroy et al. (1988, p. 355) these levels are:

1. **intrapersonal factors** – characteristics of the individual such as knowledge, attitudes, behavior, self-concept, skills, etc. This includes the developmental history of the individual. *[Here, a student, a parent, a teacher]*
2. **interpersonal processes and primary groups** – formal and informal social network and social support systems, including the family, work group, and friendship networks. *[Here, a student-teacher, parent-teacher, or teacher-teacher relationship plus larger units such as the family, an IEP team]*
3. **institutional factors** – social institutions with organizational characteristics, and formal (and informal) rules and regulations for operation. *[Here, the school]*
4. **community factors** – relationships among organizations, institutions, and informal networks within defined boundaries. *[Here, school districts, non-public schools, parent advocacy groups]*
5. **public policy** – local, state, and national laws and policies. *[Here, laws and policies concerning education and special education, including the IDEA]*

McLeroy (1988), who was concerned with health promotion through behavior change, favored an ecological model that acknowledged the reciprocal influence of individual action and social environments: People (and groups of people) act on systems and systems act on them. A consideration of multiple levels of action, meanwhile, opens the possibility of effecting desired change through intervention at any or all of them. In the current study, our interest was positive outcomes for autistic students with a focus on the first two levels: individual parent, student, and especially teacher characteristics and quality of dyadic relationships (student-teacher, parent-teacher, and teacher-teacher). In a setting where autistic students were being sent for an education and “related services” (such as speech therapy), what made key relationships positive, nurturing, or collaborative? What made them negative, toxic, or rejecting? Note that larger systemic issues linked to the third, fourth, and fifth levels (e.g., school culture, federal policies) are beyond the scope of the current study although certain aspects of these will be discussed when necessary to provide context.

Before continuing, it is important to acknowledge the positionality of the researchers regarding the study’s topic. The first author is the parent of a young adult on the autism spectrum and the second author has an autistic sibling. We chose not to reveal this to interviewees so that the focus of our encounters was on their journey with autism, not ours. We are aware that our experiences as family members of autistic individuals may have influenced how we approached this effort. Remaining reflexive throughout, we strove to ensure that any disadvantages of this “insider” status (e.g., bias) were far outweighed by the advantages (e.g., a deeper understanding of the culture under study) (Holmes, 2020). The final three authors were research assistants with no personal connection to autism.

## Participants

To summarize, participants in this study consisted of 35 parents of YAAs, 12 YAAs, 11 autistic teens, and 19 educators. To participate, parents of YAAs had to have an adult child with a current autism spectrum disorder (ASD) diagnosis who had left high school within the past 15 years. Parents were recruited through autism advocacy organizations’ social media sites and newsletters as well as fliers posted at autism-focused community programs such as social skills classes where they might drop off and wait for their YAA. Eventually, participating parents were asked to refer other qualifying families to the study.

Parent interviews were conducted in person by the first author and lasted approximately 90 min. Twelve of the YAAs and 11 teens from the SPARK-related study took part in their own separate interview. (Teens’ parents were interviewed for a separate effort but were not part of the current project.) Interviews with parents and YAAs were conducted in person; those with teens were conducted by phone (55%) or virtually by WebEx (45%). These lasted an average of 30–45 and 20–30 min, respectively, with length based on the interviewee’s interest in the process. The first author interviewed all YAAs in person at their home or an alternate location of their choice; the first author or research assistants (LF and AI) interviewed the teens. Both YAAs and teens were offered an interview only after a parent indicated they thought their child would be interested in taking part in one. Barriers to participation cited by parents of both groups included being nonverbal, being intellectually disabled, being disinterested, or being too anxious to take part in an interview. Family, YAA, and teen demographic and clinical data are presented in Table 1. Note that information on YAA and teen cognitive level and co-occurring conditions is based on parent report.

**Table 1:** Parent and young autistic adult (YAA) or teen characteristics

Parents interviewed^a^	*n* = 35		
Age of primary contact parent (SD)	54.7 (4.4)		
Parent age range (yrs)	48–65		
Parent interviewed			
Mother only (%)	26 (74%)		
Father only (%)	1 (3%)		
Couple (%)	8 (23%)		
Family income^b^			
$25K - $49,999 (%)	2 (6%)		
$50K - $74,999 (%)	2 (6%)		
$75K - $99,999 (%)	2 (6%)		
$100K - $149,999 (%)	12 (34%)		
$150K or more (%)	17 (49%)		
Primary contact parent highest education			
Some college or less (%)	2 (6%)		
Bachelor’s degree (%)	15 (43%)		
Master’s degree (%)	11 (31%)		
Doctoral or professional degree (%)	7 (20%)		
	All YAAs^a^	YAAs interviewed	Teens interviewed^c^
**Autistic individuals**	*n* = 36	*n* = 12	*n* = 11
Age (SD)	23.1 (3.3)	22.8 (3.6)	15.5 (0.7)
Age range (yrs)	19–31	19–31	15–17
Gender			
Male (%)	29 (81%)	10 (83%)	9 (82%)
Female (%)	7 (19%)	2 (17%)	0 (0%)
Nonbinary^d^ (%)			2 (18%)
Race			
White (%)	30 (83%)	10 (83%)	8 (73%)
Black (%)	4 (11%)	2 (17%)	2 (18%)
Other (%)	2 (6%)	0 (0%)	1 (9%)
Ethnicity			
Hispanic (%)	2 (6%)	0 (0%)	1 (9%)
High school completion or plan			
Diploma (%)	26 (72%)	12 (100%)	8 (73%)
Certificate (%)	10 (28%)	0 (0%)	2 (18%)
Undecided (%)	N/A	N/A	1 (9%)
Co-occurring conditions			
Intellectual disability (%)	14 (39%)	3 (25%)	2 (18%)
Seizures or epilepsy (%)	7 (19%)	3 (25%)	3 (27%)
ADD or ADHD (%)	18 (50%)	9 (75%)	5 (46%)
Anxiety (%)	24 (67%)	7 (58%)	7 (64%)
OCD (%)	10 (28%)	3 (25%)	0 (0%)
Depression (%)	13 (36%)	5 (42%)	6 (55%)
Type of high school currently attending			
Regular public (%)			8 (73%)
Other public^e^ (%)			3 (27%)

^a^ 35 families were interviewed, one with two young adults

^b^ Does not add to 100% due to rounding

^c^ Autistic teens were included to enhance autistic voices; their parents are not represented in the current study

^d^ “Nonbinary” was not offered as a choice in the Parent/YAA study

^e^ Includes Non Public School (NPS), State-sponsored Online School, and Home Hospital

The 19 educators who took part in this study were from the same mid-Atlantic area as the parents and YAAs but had no relationship with these participants. They were simply serving individuals on the autism spectrum in a variety of roles including general educator, special educator, “specials” teacher (music, art, physical education), occupational therapist, and paraprofessional. (See Table 2.) As a group they had served students across all possible grades from pre-school to extended high school (usually serving intellectually disabled students ages 18–21), and in a number of settings: public neighborhood school (where special needs students were included near or with typical students), public separate day school (serving only special needs students), and non-public school (also serving a population of students with disabilities). An exclusion criterion for educators was having experience only in private schools as our interest was in the challenges faced by those embedded in settings in or adjacent to the public school system. To recruit educators, fliers were distributed by email to people in the authors’ networks as well as through autism advocacy organizations’ social media sites. There was also a “snowball” effect as teachers told other teachers about the project. Interviews averaged 60 min and were conducted by the first and second authors in person in the educator’s home or another preferred location such as a public library meeting room.

**Table 2:** Educator characteristics

Educators participating	*N* = 19
Age (SD); range: 24–62	44.2 (12.6)
Educator gender	
Female (%)	18 (95%)
Educator race	
White (%)	17 (90%)
Black/African American (%)	1 (5%)
Other (%)	1 (5%)
Educator ethnicity	
Not hispanic (%)	19 (100%)
Education level	
Some college (%)	1 (5%)
Bachelor’s Degree (%)	3 (16%)
Master’s Degree (%)	13 (68%)
Doctoral or professional degree (%)	2 (11%)
Years experience teaching (SD); Range: 2–31	15.4 (8.1)
Years experience teaching autistic students	
1–5.99 (%)	4 (21%)
6–10.99 (%)	4 (21%)
11–15.99 (%)	3 (16%)
16+ (%)	8 (42%)
Primary role when working with autistic students	
General educator	4 (21%)
Special educator	11 (58%)
Other (“Specials” Teacher, OT/SLP, Paraprofessional)	4 (21%)
Settings in which ever taught^a^	
Public neighborhood school (%)	17 (90%)
Public separate day school (%)	6 (32%)
Non-public school (%)	3 (16%)
Grades ever taught^a^	
Pre-K/Kingergarten (%)	11 (58%)
Elementary (grades 1–5) (%)	10 (53%)
Middle (grades 6–8) (%)	8 (42%)
High school (grades 9–12) (%)	13 (68%)
Extended high school (age 18 up to age 21) (%)	6 (32%)

^a^Participants could select multiple categories so will not add to 100%.

All interviews, including those conducted with the teens via phone or WebEx, were recorded on a digital recorder and then transcribed before undergoing analysis.

Sampling was purposive in all cases. Families whose autistic child had completed high school were sought initially in the hope that they would have recent experiences to share regarding the transition to adulthood. Later, educators who had worked with students on the autism spectrum were needed, especially those willing to share their views about how these students were managing in the schools. (It is evident that teachers unenthusiastic about working with autistic students were unlikely to participate and are not represented.) Teens were sought for a study about research participation but also spoke about their daily lives including school. They were included in the study to enhance autistic voices regarding experiences with educators.

### Analysis

Atlas.ti qualitative analysis software was utilized to code interview transcripts. Parent and YAA transcripts were coded first. Indeed, it was their concerns surrounding the school years that led to our interest in the educators, whose interviews took place later and were coded separately (but with an awareness of themes that had emerged in the parent and young adult case). Because parents and YAAs were not asked specifically about the school years, they made fewer school-focused comments than teachers whose interviews were entirely focused on autism in the schools. For example, there were 26 parent or YAA comments referencing an unkind or unhelpful educator (made by only 16 of 35 parents and two of 12 YAAs), but 104 such comments made by 18 of 19 teachers. The teachers’ interviews were simply far more concentrated on education topics. The teens, who took part in a wide-ranging interview focused on thoughts about research participation, were similar to the YAAs. They mentioned school in passing, with only two teens responsible for 6 negative comments about teachers.

We utilized the constant comparative method as our approach to coding (Glaser, 1965; Boeije, 2002), with two independent coders marking transcripts on their own before coming together to compare and discuss until coming to a final consensus on a code. Our practice was not to code phrases and single sentences, but entire paragraphs or exchanges. This kept adjoining material together when the software pulled similarly coded material out of its original placement for review, providing context for participant comments. Ultimately, we had to decide which theme was the dominant one for any exchange or quotation. As three studies fed into this effort, it was sometimes necessary to revisit codes that were similar and/or grouped differently and now needed to be compared. The *School Individual – Good* code from the original parent/YAA study, for example, aligned well with the teacher codes *Other Educator – Good to Me* and *Other Educator – Good to Kids/Parents* though to directly compare them the two teacher codes had to be combined into one. Similarly, the teen code *Teacher – Good/Bad* had to be revisited and split into *Teacher – Good* and *Teacher – Bad* to permit comparisons.

## Results

All names are pseudonyms, and some small factual details have been changed to protect participants’ confidentiality. Note that teacher, parent, YAA, and teen views of interpersonal dynamics within school systems and between educators and families are complex, with an ebb and flow from the very negative to the very positive. (See Table 3 for a summary of emergent themes and subthemes.)

**Table 3:** The importance of educators who understand: themes and subthemes

1. “Getting Autism”
a. Caring: A critical ingredient
i. Understanding, accepting, having a bond with, celebrating
ii. Being there
iii. Advocating, facilitating
b. Knowledge and hard work: The “how” of helping
c. Vocation and reward: The “why” of helping
d. Other educators who “get it”: A source of support and affirmation
2. Teachers Who Don’t “Get It”
a. Lack of learned or intuitive understanding
b. Rigid mindset
c. Rejecting – views autistic student as not belonging
3. Student- and parent-based teacher challenges
a. Physical and psychological student-based stressors
b. Parents: Allies and adversaries in need

### Getting Autism

Understanding students on the autism spectrum, caring about them, and knowing how to shepherd them towards independence were crucial elements within an overarching theme: the importance of “getting autism.” These insights, coming from educators, parents, and students alike, may provide a guide for teachers similarly seeking to build rapport with autistic students.

#### Caring: A Critical Ingredient

Nineteen parents of YAAs, three YAAs, and 10 autistic teens described the importance of teachers who genuinely cared about autistic students as reflected in their deep understanding of these students, their availability, and their advocacy. Kaila’s mother said:

There are those people who just bond with your kid and get to know them as people and understand what they’re capable of… One guy, we were very lucky with him… the main thing was that he just cared.

Hunter, a teen whose passionate interest was anime, explained that he constantly inserted anime into class discussions. (“I bring up anime daily and whenever I raise my hand, I’m most likely going to bring up anime.”) He described how other students would groan when he raised his hand. He deeply appreciated his teacher who would take the information shared about *Death Note* or *Princess Mononoke* and say “Excellent, Hunter. Thank you for that information.” His acknowledgement of Hunter and a beloved topic clearly made a difference to this student.

Fifteen-year-old Ray clearly enjoyed the warmth and personality of some of his most memorable teachers: “My favorite quirky teacher is Miss Sanchez… My PE teacher, Miss Carlson, she is the funniest teacher.” Steve reported that his teachers loved teaching him:

Mr. Davis and Miss Fox are my favorite teachers and paras because they just love to teach me how to do math and division and reading and everything.

Peyton’s mother praised her adult son’s former case manager, saying she appreciated her focus on his strengths and the fact that she clearly liked him. She also praised his high school teachers for their availability, especially those who were “welcoming and helpful” after school, running clubs and providing extra tutoring. Peyton himself praised the teacher who ran his favorite after-school activity, calling him “a very close friend and mentor to me” who would listen and provide counsel when he was struggling. This notion of someone being there to provide support to students and parents alike was a critical aspect of caring. Dalton’s mother said:

For the schools to have people who are there, hands-on to help parents understand and give them advice… and like I said, if you’re lucky enough to get somebody like I did who will give you her home number, her cell number, you can call any time, and she’ll be there. That’s important.

Advocacy on behalf of students and families was also highly valued. Speaking of the teacher who had been available to her daughter through high school, Kaila’s mother related:

If she didn’t turn in a paper and was supposed to go and talk to the teacher about it and couldn’t quite get herself to see that teacher because she was embarrassed or shy or afraid… she just never went in. Then she’s failing the class because she didn’t turn the paper in, even though the paper was done… Mr. Nelson would kind of step in, and he talked to the teacher and he talked to Kaila and he got them in the same room and somehow we’d get the paper out from under the bed and… he’d help her get it to that teacher… He was like the glue that made it happen.

She viewed this teacher’s role as complex and essential: “helping these kids navigate and broker these conversations” when their anxiety or other issues prevented them from succeeding otherwise. Peyton’s mother similarly described her son’s case manager as able to identify the stressors and the motivators, finding teachers who were good matches for him, and suggesting school activities that might interest him. At age 23, Holt praised a teacher who intervened when bullies made obnoxious noises to disrupt his learning during class. While other instructors ignored the behavior, this teacher would not:

Excuse limit: zero. You do not do your work, he will give you the grade; he did not tolerate anybody messing with anybody, probably one of my best teachers ever.

Committed teachers likewise described caring about their students as essential to their practice. Many teachers spoke of an innate liking of children or teens on the spectrum, an intuitive understanding of their way of being, and a delight in taking on the challenge of working with them:

- Rachel: “They need to know that you love them. You’re there for them. You care about them. They’re not just an ABA child.”
- Becky: “I love my kids… The best move I ever made was taking over the autism classroom.”
- Emma: “I’ve fallen in love in with our kids… I really want to push these kids hard because I find that the more we challenge them, the more they will rise to be all that they can be. And if we don’t do that, then it’s a disservice because then they’re not equipped to do the things they can do because they don’t think they can, and that can be heartbreaking, and I just don’t want to see that anymore.”

Teachers stressed the importance of building relationships with their students the better to grasp their struggles and talents. “Because I take my role very seriously, I get to know my kids,” said Vicky. Molly also valued knowing and connecting with her students the better to serve them well:

Earlier on it’s really getting your hands dirty, trying to figure out each student, getting that buy-in, building that rapport, getting that connection with them…”.

Shawna described how, with a strong relationship as a foundation, she could ask anxious high school students to trust her:

I have seen kids that have been so far behind and thought that the world was coming to an end that have been able to catch up within a week or two. And it’s being able to look at them and say, ‘I promise you, for the next two weeks if you will work with me, we can get your grade up. You just have to believe in me.’

Some teachers stayed connected with students and families even after graduation. Rachel explained:

For my high school kids, I say, ‘Here is my number. If you need me, call me.’ Some parents do, and some parents don’t but it makes me feel good when a parent feels comfortable enough to say, ‘I’m having trouble with the day program. Could you come help?’ Of course, I would love to come help. I want them to be successful.

Teachers admitted that not every autistic student was easy to have in a classroom. Even so, many would try their best to make them feel liked and appreciated. Shawna explained that she “would die” if a child ever guessed she didn’t like them:

Even when you don’t [like them], you have to go out of your way. That kid will never know I don’t like him. He will never know. I will find some redeeming value for that child and I’m going to work on it.

Vicky described supporting her more challenging autistic students when they came to her in distress after encountering another teacher’s disfavor:

They will say to me, ‘I hate going in there. She hates me, and I know it.’ I go, ‘She doesn’t hate you. She just doesn’t get you. I’m the lucky one who gets to hang with you.’ I always say, ‘It sucks for them, because I’m the one who gets you. I’m the one who gets to have fun.’ It’s not fun. It’s awful. But I tell them it is.

#### Knowledge and Hard Work - The “How” of Helping

Caring mattered but by itself was not sufficient. Teachers also needed knowledge to be effective at educating autistic students, and a willingness to work hard at it. Fifteen-year-old Liza, who said her teachers were “doing their best,” was quite aware that she needed a specific type of help:

I have to get step-by-step instructions, have a step-by-step recipe. I cannot figure something out on a lot of times. I can figure some out, but at the same time if there are directions, I need exact directions. If someone does not tell me exactly what I am supposed to be doing, I am going to absolutely panic.

Teachers understood that needs were unique and heterogenous. Maria spent a lot of mental and emotional energy trying to ascertain just the right intervention for each student:

I am the type that I keep digging until I find my answer. You know? I lay in bed at night. I am like, okay, why is that student doing this? What am I doing wrong? What have I not tried?

The determination to try multiple approaches, to unlock what a child who might be hampered by cognitive, communication, or mental health difficulties needed, was the mark of these teachers. Vicky explained:

Even though they’re not verbally always saying something, they’re saying something. Their body and what they’re doing, they’re writing or they’re drawing, or they’re shutting down with their head on their desk… Whatever it is. I recognize it. I back off, or I come close, or whatever it is I feel they need.

Claudia stated that she employed a child-centered, total communication approach, rejecting some adult-imposed aspects of current applied behavioral analysis (ABA) practice:

We did really start focusing on how to communicate and how to start reciprocal anything – reciprocal communication, reciprocal play, my turn, your turn. We changed our warmup to include this choice board idea and then ‘Who wants a turn?’ and can we get a hand raise? Can we get an eye gaze? Can we get an affect change? I’ll take anything.

Emma emphasized the importance of patience, structure, and using the routine-loving nature of autistic students to advantage:

It’s making sure that there’s structure, expectations, daily protocols, weekly routines, everyday agenda book checks, all these things that I’ve had to incorporate into my program to equip these kids to do the best they can do… That’s one thing about kids with autism: when they have a routine, and they understand, ‘That’s my routine,’ they’re good. They’ll walk to the ends of the earth doing that routine.

Rosie said that some of her school’s most challenging autistic students calmed down when around her, something she felt good about. This, plus the use of what she called “empathetic strategies,” led to her success helping students having meltdowns. Becky also used approaches based in empathy like meeting the needs of her anxious high school students by establishing scheduled time to address low grades or missing assignments:

They want to do well but they just feel like they’re drowning… When the tears start flowing… they’ll say to me, ‘Can I just have a make-up day?’, and I’m like, ‘I think that’s a great idea.’ We sit down and we make a plan.

Teachers were often eager to share their knowledge, demonstrating for colleagues that a child thought to be limited could make strides the other educators had not imagined possible. As Jasmine put it: “I think we have to stop telling the kids what they can’t and will never achieve and find ways to help them achieve it.”

#### Vocation and Reward - The “Why” of Helping

Some teachers described teaching students on the autism spectrum as their calling. Molly, whose sister was “on the spectrum a little bit,” had witnessed her parents struggle to navigate the educational system and described helping others through it as a “huge motivator.” In addition, valuing her students and advocating for them were central:

They make my day. I could have a bad day and bad morning and I walk in, and you have the student that says, ‘Hey, you’re here, I missed you.’ ‘You know what, dude, you’re awesome.’… It definitely gives a lot of purpose, and obviously it drives me. I want to do what’s best for them… I think my big thing is membership because I hate the fact that many of these students are just pushed in the back of a closet and the back of a room and nobody sees them.

It was deeply satisfying when all the work to connect, to find the right approach, and to patiently implement it led to success. Molly called these “lightbulb moments.” Dominique explained:

It’s just that joy of them figuring it out and realizing that they don’t have to scream…. Then once the communication comes together, the behaviors kind of go away.

There was even more satisfaction when something worked after other attempts had failed. Maria described giving up on the prescribed phonics method of teaching reading when it became evident it was not working for her fourth-grade autistic student with severe apraxia:

I took a different approach with him. I stopped trying to push phonics down his throat. I was like, ‘You know, he has had phonics all these years. He is not getting it.’ I tried a whole-word reading program. By the end of my two years with him, I had him reading three and four sentences together.

#### Other Educators Who “Get It”: A Source of Support and Affirmation

The teachers warmly acknowledged fellow educators who, like them, understood autism and provided much needed support to them, their autistic students, and the students’ families. That support might take the form of encouraging a student who was feeling defeated, sharing a student’s accomplishment with a parent who rarely heard any praise for their child, or brainstorming solutions when a colleague had no idea how to address a challenging situation involving a student on the spectrum. Descriptions of these helpful individuals, from paraprofessionals to administrators, included phrases like “they just get it,” “they just care,” “they took the time to learn what they needed to learn,” and “they have an innate understanding.” Speaking of a colleague who connected with one of her students, Jasmine said:

Her math teacher would come in and sit down with her on his lunch. He would talk to her like she was responding, and then eventually one day she started talking back. A lot of times people just give up too quickly.

These educator-allies provided information, resources, and encouragement. Ideally, dedicated teachers sought out others of like mind, supporting each other and making each other better. Dominique said it was good to talk to others in the same boat, “just to know that you’re not doing this alone, everybody is having a tough time and it’s great to share our good moments.”

Unfortunately, teacher statements about unhelpful colleagues outnumbered those about supportive ones by nearly three to one.

### Teachers Who Don’t “Get It”

Just as caring teachers lived long in the memory of students and parents, so did uninformed, unaccepting, or even cruel ones who they viewed as having done lasting harm. Educator-participants described some of their least favorite co-workers in similar terms. Common elements were a lack of understanding; a rigid approach; an unkind interpretation of behavior (e.g., not overwhelmed, but lazy); and a rejection of autistic students who were viewed as not belonging in their classroom or school.

#### A Lack of Learned or Intuitive Understanding

That many educators and administrators lack an understanding of autism was mentioned repeatedly. Speaking of her daughter’s high school experience, Amber’s mother shared:

If she was not compliant, they would not let her have lunch with her friends. And then they wanted her to interact with people in her own class, not in lower classes. And I am saying to myself: ‘Do you not want the kid to connect and have relationships?’

Kaila’s mother would try to ease her daughter’s way by explaining to the teacher what she needed. The response she received was not helpful:

I came up to the teacher when we were heading off to the field trip and I said, ‘You know, Kaila might get a little anxious about going on the boat.’ And she rolled her eyes at me. It’s like, what do you not understand about accommodating disabilities and how that’s part of your job?

Teachers likewise pointed to a lack of understanding on the part of colleagues:

- Rachel: “When I worked in a regular school in a life skills classroom, the principal didn’t understand. The vice-principal didn’t understand… Nobody understood.”
- Lila: “Some of the teacher’s don’t know what IEPs are. They don’t understand autism.”
- Chris: “There are a handful of people in this building who… I will use the term ‘get it.’ There are a handful of people in my *prior* building that ‘get it.’”.

Some commented that child behaviors like screaming made it difficult for their colleagues, calling them “not comfortable” or “not confident.” Some felt their colleagues’ limited understanding translated into a lack of respect for their work and for their autistic students’ potential. Dominique wanted administrators to “understand what we do and what these kids can do,” while Marion felt misunderstood by uninformed colleagues:

If someone is having a challenging behavior, there are certain things that we ignore in an effort not to reinforce them. But then the general educator just wants that behavior to stop. And they look at you like, ‘Why aren’t you doing anything?’ But you’re actually doing what you’re supposed to be doing… I think we’re kind of looked down upon.

According to Jasmine, autistic students were quite aware when someone disliked them:

All of my students have teachers that they want to work with, and they have teachers that they would rather have an unsafe behavior to escape working with. It’s usually my staff that are a little hesitant or not as knowledgeable in the field… That’s a little frustrating at some times because people think our kids aren’t capable, and they’re fully capable. You just need to meet them where they are.

#### Rigid Mindset

Parents fretted about teachers who interpreted what their child or teen did in the most damaging way possible. Calling 10th grade “a disaster,” Griffin’s mother explained:

The English teacher…was saying, ‘He can do the work. He’s choosing not to.’… I said, ‘It’s because he doesn’t get it. English for Griffin is almost a joke, it is almost like English as a second language…’ She just felt it was all effort related.

Teachers shared similar instances where colleagues interpreted undesired behavior as willful. If an autistic student wasn’t looking at the teacher, they were not paying attention. If they were making noise, they were “attention seeking.” Usually, these assumptions were wrong and so was the response, leading to an escalation of student behaviors and battering self-esteem. Lila reported how a bright autistic student with anxiety was misunderstood when he showed clear signs of distress:

I send out e-mails to the team leader. These are the behaviors I’m noticing with this kid. He’s anxious. He’s not performing, he’s just not working anymore… But his case manager is like, ‘Here are his new objectives, and this is what we’re doing,’ and she’s trying to measure them. I’m like, ‘He has shut down…You need to try another approach…’.

Teachers who “get it” expressed impatience with teachers who did not. Molly described a teacher who was adamant that her student stop stimming:

Well, it doesn’t need to stop. In fact, I’m sorry they’re tapping their pen and it irritates you, but it’s not going to end the world. That’s just what they need.

Debbie agreed. She was trying to increase acceptance to fight these negative attitudes:

As soon as they see this kid, they want to send him out of the room because he is spinning in the background. I am like, ‘Okay. Is he disturbing anything? If he needs to spin for a little while to get that movement and he is still in the classroom absorbing what the teacher is saying, does he really need to leave?’

Providing a fitting summary, Jasmine declared: “They say our students are rigid, but I find that our kids are more flexible than some of the teachers.”

#### Rejecting – Views Autistic Student as Not Belonging

Parents told painful stories about their child—and, by association, their family—being unwanted by a school.

- Mack’s mother: “This principal had very explicitly told anyone and everyone who was to deal with my son she did not want him in the building, and they were to basically do everything humanly possible to make it so that he had to leave her building as soon as possible. She did not want ‘those kids’ in her school.”
- Dalton’s mother: “I realized all he was doing in school was coloring, just doodling and really making noises. The teacher would get upset because she didn’t want this child in her room, and so it was a very hard time for us.”
- Antonne’s mother: “She’s not helping my child. She understands that there is something wrong but she’s not doing anything to help him. He’s just kind of sitting in the corner and she’s teaching the other kids.”

Students were not immune to such dynamics. A teen named Will described a person who sat at the sign-in desk at school. He was certain that she did not like him. She would make him sit close to her because he was getting distracted, putting him in an “exposed” spot that took a toll on him. He also felt disliked by one of his middle school teachers, and the reason for her distaste was clear. “She did not like me. She did not like that I did not learn like other people.” Liza also discussed feeling disliked and unwanted:

The lady in the Guidance Counselors office at the desk where you sign in, she made the environment there feel very hostile. She made me feel very unwelcome there… She would always give me a hard time when I wore noise cancelling headphones.

In a similar vein, teachers remarked that some of their colleagues did not want autistic students with any interfering issues in their classrooms. Saying some teachers get it and love it, some get it and cannot handle it, and some do not get it at all, Molly stated that “a lot of them believe autistic students shouldn’t be in their room.” Maria gave an example:

Sure, I see some inattention. Maybe there is a little bit of social awkwardness, but nothing off the radar. These teachers are telling me, ‘He does not belong here. He needs a different setting.’ I am like, ‘Why?!’ They actually are referring him to a non-public school at this point… because they are so averse to making the effort on their side to make it work.

Alexis, who worked in a separate day school for students with significant challenges, explained that she resolved a supposedly out-of-control aggressive student’s behaviors in five days which meant “he probably didn’t need to come to me.” She described how a special education teacher in a general education school sent her multiple students he clearly did not want in his classroom:

Each time I went to the school to observe and consult on these students it was abundantly clear that the problem was not with the students, it was with the teacher, who was obvious in his dislike of each of these students… His complaints about the students’ behavior were always stated in front of the students and many of the complaints were with changes he had to make to his classroom routine… The part about this that broke my heart was that we were sending students to more restrictive environments even though we knew that the teacher was the problem.

Maria felt that autistic students were “nit-picked on” about their behaviors even more than their typical peers and to the point that it was detrimental. Rosie related an incident where a highschooler who “does very obvious stimming” and sometimes curses without malice was “taken out in the hallway and yelled at unmercifully.” Jasmine reported an incident where a bright, nonverbal middle school student slightly varied an assignment he was working on in a “specials” class. His teacher responded by destroying the student’s work:

This teacher was bullying him because she thought he couldn’t speak. The student shut down and just walked to the back of the room and stood with his back to this teacher. This teacher was screaming at him, belittling him. No consequence to the teacher….

Teachers were angry and upset when colleagues were unreceptive to what they knew about autism, to programs they had fought hard to develop and implement. Lila said:

I’m pissed, I’m really pissed. I hear the student’s frustrations and I see it… It’s like, ‘Okay seventh grade, what are you guys doing about this kid? What do you mean, nobody else has said anything about this kid? What do you mean? What’s your problem? What’s *my* problem? My problem is you’re not listening to me…’.

Alexis would work with students, including very aggressive or out-of-control ones, to build a relationship and help them progress towards communication, self-regulation, and relationships, often at the cost of being bitten and bruised. She felt defeated when others refused to continue the program she had put in place:

I had to keep telling people. I’m not special. It’s not me. I do their program. Once we find a program that works for them, just run the program. But what happens is I develop a program for a student to be successful, move to another classroom and they wouldn’t run the program. They just wouldn’t. I don’t know why… And then the student just crumbles and everything goes down.

### Student- and Parent-based Teacher Challenges

When teachers of autistic students did not receive support from colleagues, it added to their stress. Of course, they faced some additional challenges working with their vulnerable students and their students’ often worried parents. Those challenges varied with the age of the student, the severity of the autism, and where the parent and student were in their autism journey.

### Physical and Psychological Student-based Stressors

When a student lashed out physically, teachers understood it was not personal, but bore the brunt nevertheless. They described being bitten, kicked, scratched, and bruised yet acutely aware that they had to maintain calm or escalate the situation further. The older and bigger a student was, the harder this became. Rosie remembered a “husky” student who had major meltdowns in middle and high school. One of his teachers had to have back surgery after he threw her against a chair. Becky recalled a ninth grader who left her with “a scar on my arm where she bit me all the way to the bone.” Rachel explained:

Our personal stuff is destroyed because, you know, all the teachers buy their own stuff… I have had some kids that are really dangerous. They will hurt you… I have been sent to the hospital… It’s not because they’re vicious or horrible people. They just lose control of themselves. They can’t stop themselves… We have a boy here, he punched me in the mouth this summer.

Alexis felt it was worth bearing some of the initial physical attacks in order to gain a student’s trust. She viewed it like being a lifeguard willing to endure a drowning person’s thrashing panic, saying “I’m willing to do it. Go ahead, I might get a little bloody. It’s okay. I heal.”

Whether teachers had students in crisis or students much easier to guide, they described emotional impacts including feelings of inadequacy or guilt when they felt they hadn’t done enough. Shawna explained:

I have to look at myself in the mirror… Did I do everything I possibly could for that child? No…? And I can’t just shake it off… I have been known to call parents at night. I will do it on the weekend, too.

A lack of knowledge and support made it especially hard for new teachers. Julia, a young teacher just finishing her first year with autistic pre-schoolers, confided:

A lot of the behavior things are my toughest challenge… I’m like ‘I want to help you so bad but I don’t even know what to do right now…’.

In brief, there was no denying the fact that these dedicated teachers faced considerable challenges with varying levels of expertise and support.

### Parents: Allies and Adversaries in Need

Parents of autistic students may be anxious, angry, or otherwise under pressure as they face key moments in their autistic child’s school career. Teachers of these children have an entire second job: understanding parent perspectives while educating and collaborating with them. Many worked hard to build relationships with parents. Becky spent her early mornings maintaining a contact log that reflected her daily texts and emails to parents, something worth doing because having good rapport “is huge.”

Molly explained that parents weren’t always easy to work with. Some of them were struggling with poverty, drug addiction, or other issues in addition to having a special needs child. Frequently, the same empathy and patience required to teach children on the spectrum was needed to work with their parents. As Claudia noted, parents were often “pretty desperate people” who were experiencing “their own trauma and struggles.” Understanding what parents were going through was critical. Rachel, who had worked in a home-based program for a time, said:

When I saw what parents have to deal with day in and day out in their home, it kind of softened me a lot… I think that should be part of your education, not just working in schools, but you should work in a home program.

Of course, conflict did arise, and from many sources. Anna mentioned that parents of younger children were especially prone to denial, while Dominique spoke of the needs of these parents for education and resources as they struggled to learn how to support their child’s progress. Shawna struggled to get anxious parents to let go and trust the interventions that would improve behaviors and support eventual independence even if they pushed both child and parent out of their current comfort zone:

When they are still trying to be helicopter parents, you’re like, ‘Please let me be school mom.’ It may take anywhere from a full year to two, but eventually they catch on and it’s like, ‘My life at home is a lot better.’

Jasmine also worked to get parents to “understand the strategies that we use within our classroom so they can mirror them at home and see the same success.” She remarked on parent fears of pushing the envelope by letting their child go out into the world a bit more, worried about meltdowns they didn’t have the tools to address. But that reticence meant a curtailing of experience and growth as when a mother insisted her high schooler stay in his “special class” all day instead of going to regular classes he wanted very much to attend. Supports like a card that permitted him to have a break whenever needed were put in place, the mother yielded, and the student had a much expanded and successful high school experience. Brianna stated:

Sometimes as parents, when we have a special needs kid, sometimes our fears and inhibitions can be projected on the child more so than what the child is actually experiencing and sometimes we may make the case worse than better.

Alexis similarly argued to parents and educators alike that the answer to student issues was not always a one-on-one aide:

Do you understand that sets them up for failure in the adult world? They’re not going to be successful and they’re not going to have one-on-one in the adult world… My perspective has always been how to make them as independent as I possibly can.

A common way of addressing the stress of conflict with parents was to recognize that all parties wanted what was best for the student. As Molly said:

It’s all coming back to the fact that everybody, regardless of what side of the table, is trying to do what’s best for the child. And that, I think, has helped with my own anxiety of coming to those difficult meetings.

## Discussion

Parents and autistic students shared experiences, good and bad, from these students’ time at school. Teachers likewise shared their views on what was going well or poorly for autistic students and themselves. Families and YAAs were interviewed when the YAA was 19–31 years old, often looking back on experiences in the schools that had occurred years ago. Teachers, who were interviewed four years after families, reflected on working with autistic students across their careers. Teens were interviewed a year after that and while still in high school. Irrespective of these varied timelines, there was a convergence of views regarding the value of teachers who “get” autism vs. those who do not. (For a summary of themes and subthemes, see Table 3.)

Teachers who were remembered with fondness and gratitude by parents, YAAs, and teens resembled the ideal outlined by dedicated educators of autistic students. This ideal went beyond a description of superlative teachers in general –those who “not only get outstanding academic results, but also provide a more engaging learning experience for students” (The New Teacher Project, 2012, p. 2). In addition to this, they were caring and present, sought out knowledge to better understand their autistic charges, advocated for them, and modeled for others how to understand and work with them. Some claimed an inherent ability to connect with their autistic students. They were willing to puzzle out what was driving an unwelcome behavior and to try different approaches. They were not frustrated but animated by the challenge. They supported parents, whose wellbeing and role in their student’s success they viewed as partly under their purview. They cared about long-term outcomes which made them willing to put up with short-term inconvenience—like a student’s acting out—in the service of a student’s future quality of life and increased independence.

Likewise, families’ and students’ descriptions of educators who were unwelcoming, uninformed, or even cruel closely resembled those of teachers discussing disagreeable colleagues. These less-than-ideal teachers had little knowledge about autism and no intuitive understanding of it. They were rigid in their mindset, applying whatever lens they used to interpret typical students’ behavior to the behavior of autistic students. A child avoiding eye contact was inattentive, not uncomfortable; a student covering their ears and making noises was attention-seeking, not overwhelmed by sensory input. These educators often resisted changing their viewpoint or practices. They frequently disliked their autistic students. They sometimes expressed the view that these students did not belong in their classroom or school.

It is important to acknowledge that the binary narrative of our participants likely masks a more complex and messy reality. Still, it is useful to draw lessons from these “good” vs. “bad” exemplars. There are two sets of characteristics common to our teachers who “got autism”: knowledge, skills, and experience on one hand and a collection of intangible personal qualities like empathy and commitment on the other.

Knowledge of autism was essential when it came to teaching these students. Nearly 80% of the participating teachers had graduate degrees in education, and about 80% had at least six years of experience working with autistic students. 68% had attended an autism-focused professional development event, 47% had attended an autism-focused conference, and 42% had taken an autism-focused course. This was important since Maria said her Master’s program in Special Education focused more on how to write IEPs than how to teach autistic children; Lila estimated that only 10% of staff and educators in a school understand autism; and Debbie declared it is not safe to assume that teachers have “knowledge or background about students with ASD.” Their comments dovetail with literature that shows general education teachers in inclusive settings often lack training that would make them successful with autistic students, including a better understanding of autism itself (Able et al., 2015). In one study, general education teachers wished for, among other things, didactic strategies and increased confidence working with students on the spectrum (Van Der Steen et al., 2020), while in another, teachers trying to implement evidence-based social skills interventions for autistic students felt wholly unprepared (Silveira-Zaldivar & Curtis, 2019). On the positive side, unmet training needs are something it is possible to remedy.

Teacher-participants certainly viewed the knowledge that they went out of their way to attain as crucial to their understanding of students on the spectrum and hence their practice. Training is not everything, however. For example, one study investigating the quality of student-teacher relationships for young autistic children found ASD trainings for teachers were unrelated to student-teacher relationship quality (Caplan et al., 2016). Teachers’ personal characteristics such as emotional intelligence (Pizzo et al., 2018) or engagement and self-efficacy (Love et al., 2020) are also related to teacher quality. In total, these are known as “teacher effects.” These are similar to “therapist effects,” meaning that even when two therapists are following the same treatment protocol patient outcomes may vary with differences attributed to the “facilitative interpersonal skills” of the therapist (Anderson et al., 2009, p. 755). Here, it is the person-of-the-teacher that is thought important in addition to any specific instructional technique or evidence-based practice. Konstantopoulos (2012) explains:

The evidence from both experimental and observational data has suggested that there are substantial differences among teachers in their ability to produce achievement gains in their students. However, the teacher characteristics that are typically observed, such as education, experience, licensure, or salary have not been consistently related to student achievement… This indicates that the most important teacher characteristics are usually not observed because they are difficult to measure. (p. 45)

Many of the most meaningful traits of the teachers who “got autism” certainly qualify as difficult to measure. Descriptions of teachers’ commitment to their autistic students, including after school or even after graduation, were compelling. So was their ability to patiently get to know their students, adapt to meet their needs, and spend sleepless nights thinking about how to reach them. They also displayed qualities mentioned by Bardach et al. (2022) in their review of teacher psychological characteristics impacting teacher effectiveness, including self-efficacy, enthusiasm, and emotional intelligence (EQ). Except for those who were very new to teaching autistic students, there was a clear sense of confidence in their ability to effectuate student progress (self-efficacy). There was a sense of connection and joy in teaching these students (enthusiasm). There was also an ability to read and respond to students cues despite atypical communication (EQ).

The concept of “emotional labor” may illuminate some aspects of these teachers’ practice. When Hochschild (1983) originated this term, the focus was on “surface acting” (p. 37) – as when a hotel clerk manages a smile for all approaching the front desk—and “deep acting” (p. 38) –where a flight attendant is taught that she must bring her emotions in line with her smile to make it sincere. The emotion that one was expected to display or generate was dictated by “feeling rules” which “guide emotion work by establishing the sense of entitlement or obligation that governs emotional exchanges” (p. 56). Diefendorff et al. (2005) later investigated a third type of emotional labor: “naturally felt emotions” occurring in line with a worker’s own disposition and perhaps enjoyment of or pride in the role they occupy. Teachers, for example, are not like hotel clerks who must muster warmth for a brief encounter with a guest. They are forging longer-term, meaningful relationships with students and parents, often involving genuine caring or a compassion-motivated facsimile thereof (Kerr & Brown, 2016). (Recall the teacher who “would die” if a student knew she didn’t like him.) Notably, more genuine emotional connections may be protective against burnout; Yin et al. (2019) found teachers engaging in surface acting were more prone to burnout and decreased teaching satisfaction than those who expressed naturally felt emotions.

Gender is another aspect that bears mention here as female-dominated professions like teaching come with feeling rules that dictate one genuinely care about one’s students. According to Bolton (2007), this coexists in tension with a masculine model of professionalism that emphasizes accountability, efficiency, control, and data – again with an emphasis on measurement. After interviewing teachers of young children with emotional and behavioral difficulties, Bolton marveled that they managed to meet the requirements of various “management systems of control and external accountability” while offering “a relational bond to the children they are asked to educate that no performance management system or professionalization project could ever hope to capture” (p. 27).

This closely resembles the balancing act our teacher-participants described. Given a chance to discuss their daily practice, it was clear credentials and accountability mattered, but that connection and caring mattered more. Unfortunately, these elements are more difficult to capture than test scores such that teachers who “got autism” were not always rewarded. Nor, as these teachers described it, did unhelpful, unreceptive teachers bear consequences for their lack of understanding. This failure of school systems to distinguish between the best and worst teachers is where larger, systemic issues grounded in Levels 3 (school) and 4 (district) of our socio-ecological model come into play. Indeed, in Weisberg et al. (2009) published *The Widget Effect*, a report that documented the tendency for all teachers to be treated exactly the same, with little acknowledgement of the accomplishments of extraordinary teachers and little effort to remove the failing ones. Nearly ten years later, an attempt to follow up found little had changed (Kraft & Gilmour, 2017). In a similar vein, TNTP (2012) found that “irreplaceables” –that is, engaging teachers whose students made great academic progress—were often treated as expendable. Although it could take 11 hires to find one teacher of comparable quality, schools took little effort to retain them.

One is left to consider how we can create and retain more of the autism-affirming teachers described herein. What can be done to provide more autism-focused training to general and special educators? What is needed to recognize, measure, and reward the extraordinary levels of caring, compassion, and creativity displayed by these teachers? What would be the most effective way to change the views and practices of teachers engaging with autistic students in negative ways? Future research should include a deeper investigation into a broader range of teachers’ experiences, including those not currently autism-positive, to determine how they are processing what is occurring with autistic students in their schools, how to change attitudes, and how to increase skills. Exploring teachers’ and students’ experiences in different settings (i.e., within private schools) may also yield insights into promising practices. Lastly, it will be important to investigate the overlap between teacher- and student-level challenges and larger systemic ones like funding shortfalls and understaffing.

### Limitations and Strengths

This study had several limitations. Purposive sampling permitted an in-depth look at the school-based experiences of individuals on the autism spectrum and their families as well as those of committed teachers of autistic students. However, this approach left out the views of teachers less familiar with autism. In addition, all samples (family/YAA, teens, teachers) were largely white and non-Hispanic. Furthermore, each sample was interviewed at a different time point and participants were not necessarily embedded in the same schools or districts. Although the autistic teens were interviewed during the COVID pandemic, not all shared related details (e.g., whether they were attending virtual school) so some of this context is unavailable. The study also had several strengths. It took a unique approach, drawing inspiration from family and YAA school-based narratives then intentionally asking teachers of autistic students and current students themselves to share their views on how autistic students are faring in the schools. This triangulation yielded striking similarities in family, student, and educator perspectives concerning the type of teacher that is most successful with autistic students.

## Conclusion

This study illustrates that knowledgeable, caring teachers can make a lasting difference in the lives of autistic students and their families. Future research on this topic should include more diverse samples in terms of ethnicity, race, and socioeconomic status as well as a more extensive exploration of how dyadic relationships between students and teachers, parents and teachers, and teachers and teachers are affected by larger systems dynamics such as school culture, district practices, and state and national policies.
